# CNNArginineMe: A CNN structure for training models for predicting arginine methylation sites based on the One-Hot encoding of peptide sequence

**DOI:** 10.3389/fgene.2022.1036862

**Published:** 2022-10-17

**Authors:** Jiaojiao Zhao, Haoqiang Jiang, Guoyang Zou, Qian Lin, Qiang Wang, Jia Liu, Leina Ma

**Affiliations:** ^1^ Cancer Institute of the Affiliated Hospital of Qingdao University and Qingdao Cancer Institute, Qingdao University, Qingdao, China; ^2^ School of Basic Medicine, Qingdao University, Qingdao, China; ^3^ Oncology Department, Shandong Second Provincial General Hospital, Jinan, China; ^4^ Department of Pharmacology, School of Pharmacy, Qingdao University, Qingdao, China

**Keywords:** arginine methylation, deep learning model, amyotrophic lateral sclerosis (ALS) pathway, CNNArginineMe, machine learning

## Abstract

Protein arginine methylation (PRme), as one post-translational modification, plays a critical role in numerous cellular processes and regulates critical cellular functions. Though several *in silico* models for predicting PRme sites have been reported, new models may be required to develop due to the significant increase of identified PRme sites. In this study, we constructed multiple machine-learning and deep-learning models. The deep-learning model CNN combined with the One-Hot coding showed the best performance, dubbed CNNArginineMe. CNNArginineMe performed best in AUC scoring metrics in comparisons with several reported predictors. Additionally, we employed CNNArginineMe to predict arginine methylation proteome and performed functional analysis. The arginine methylated proteome is significantly enriched in the amyotrophic lateral sclerosis (ALS) pathway. CNNArginineMe is freely available at https://github.com/guoyangzou/CNNArginineMe.

## 1 Introduction

Protein arginine methylation (PRme) is a common post-translational modification (PTM), which plays a crucial role in pre-mRNA splicing, DNA damage, signaling, mRNA translation, cell signaling, and cell fate decision (Blanc and Richard, 2017; Kumar et al., 2017; Wang S. M. et al., 2019; Abe and Tanaka, 2020; Parbin et al., 2021; Scopino et al., 2021). Arginine contains five potential hydrogen bond donors favourable for interactions with biological hydrogen bond acceptors (Yang and Bedford, 2013). Types of arginine methylation include ω-N^G^-monomethyl arginine (MMA), ω-N^G^, N^G^-asymmetric dimethylarginine (ADMA) and ω-N^G^, N^G^-symmetric dimethylarginine (SDMA). A family of nine protein arginine methyltransferases (PRMTs) catalyzes the formation of MMA, ADMA, and SDMA in mammalian cells (Bedford and Clarke, 2009; Yang and Bedford, 2013; Poulard et al., 2016). PRMTs are classified into three groups of enzymes (types I, II, and III) according to their catalyzed types of methylations. All of them produce MMA, and type I PRMTs (PRMT1, PRMT2, PRMT3, Carm1/PRMT4, PRMT6, and PRMT8) form ADMA, while Type II PRMTs (PRMT5 and PRMT9) form SDAM, whereas PRMT7 is the only Type III enzyme, exclusively catalyzing the formation of MMA (Poulard et al., 2016). Arginine methylation has regulatory effects on various physiological processes and pathological conditions; dysregulation of the enzymes is associated with several diseases, such as cancer (Boulanger et al., 2005; Covic et al., 2005; Ratovitski et al., 2015; Fedoriw et al., 2019; Guccione and Richard, 2019; Szewczyk et al., 2020). Therefore, it is essential to accurately predict methylation sites to understand PRme molecular mechanisms.

Traditional experiments used to identify methylation sites—such as mass-spectrometry, methylation-specific antibodies, and ChIP-Chip, are labour-intensive, expensive, time-consuming, and require a high level of technical expertise (Wilkins et al., 1999). With the increase of the identified PRme sites, computational methods have emerged as an efficient strategy to complement and extend traditional experimental methods for PRme site identification.

Eleven computational predictors have been built to predict arginine methylation, including nine machine-learning models and two deep-learning models. In the machine-learning models, MeMo was constructed using sequential features (Chen et al., 2006). Shao *et al.* incorporated a support vector machine (SVM) algorithm with a Bi-profile Bayes feature extraction method (Shao et al., 2009). The model MASA combined the SVM algorithm with protein sequences and structural characteristics (Shien et al., 2009). The model PMeS was based on an enhanced feature encoding scheme (Shi et al., 2012). The predictor iMethyl-PseAAC was formed by incorporating the physicochemical features, sequence evolution, biochemical, and structural disorder information into the general form of pseudo amino acid composition (Qiu et al., 2014). The model PSSMe was based on the information gain optimization method for species-specific methylation site prediction (Wen et al., 2016). The predictor GPS-MSP was developed to predict different PRme types, the first model for predicting each PRme type (Deng et al., 2017). The model MePred-RF integrated the random-forest algorithm with a sequence-based feature selection technique (Wei et al., 2019). Hou and coworkers built a model to predict PRme sites based on composition-transition-distribution features (Hou et al., 2020). In the deep-learning-based models, CapsNet contained a multi-layer CNN for predicting PRme sites, which outperformed other well-known tools in most cases (Wang D. et al., 2019). The deep-learning model DeepRMethylSite was constructed with the integration of One-Hot and embedding integer encodings (Chaudhari et al., 2020). The development of these models has contributed significantly to the discovery of PRme sites.

The limitation of experimentally verified PTM data is often the main reason for inaccurate prediction. With the increase of PRme sites, it is necessary to re-investigate the predictors for PRme sites. We developed and compared several prediction models with the reported predictors in this study. We found that our deep-learning model CNNArginineMe had the best performance. Moreover, we used CNNArginneMe to predict human proteins that contained PRme sites and performed biological function enrichment analysis for these proteins using Gene Ontology (GO) and Kyoto Encyclopedia of Genes and Genomes (KEGG).

## 2 Material and methods

### 2.1 Dataset preparation

**FIGURE 1:**
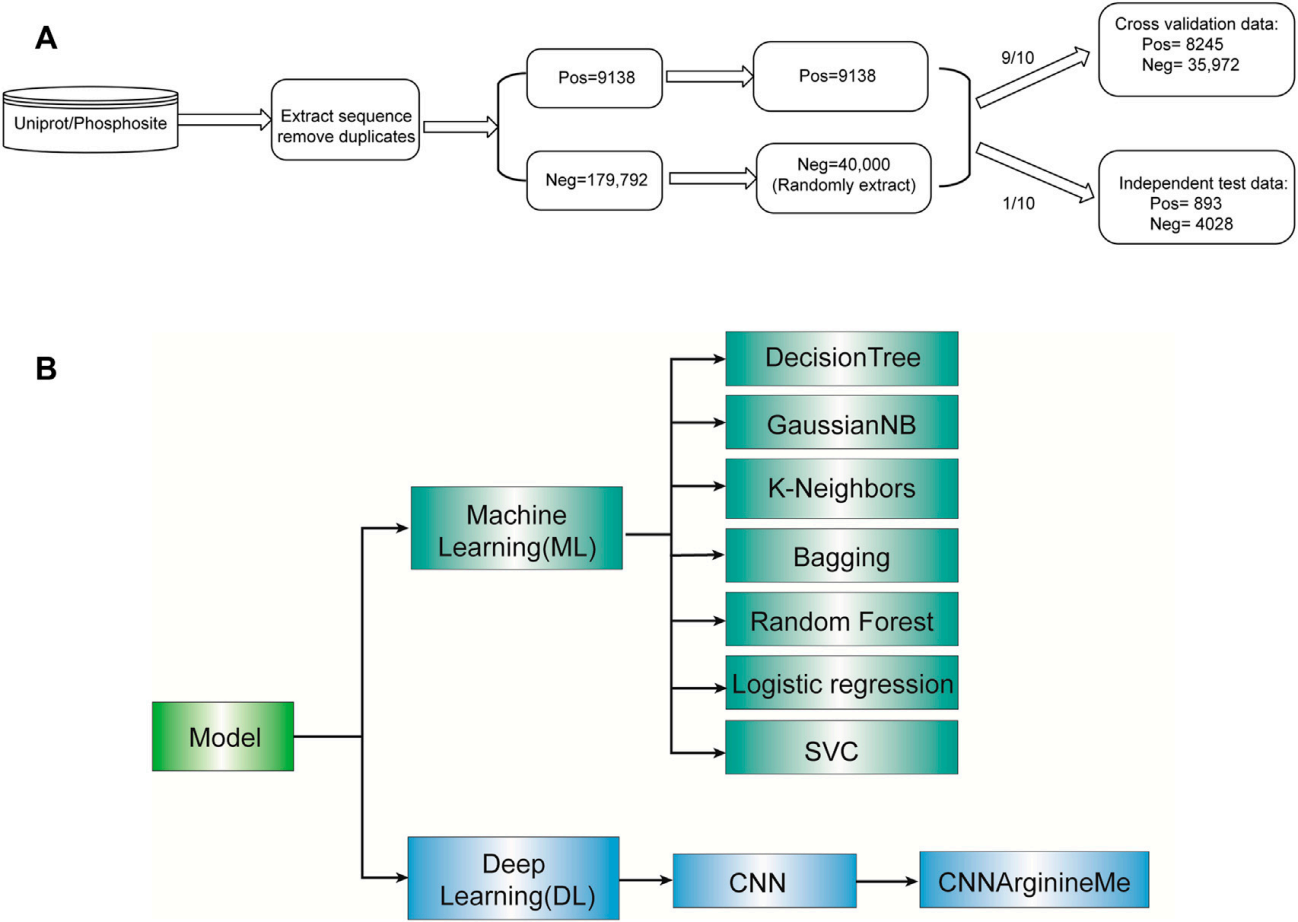
Roadmap of this study. **(A)** Flow diagram depicting data pre-processing. **(B)** The algorithms selected for model construction.

Figure 1A shows the construction procedure of the dataset. Specifically, we extracted the human PRme-containing proteins from phosphositePlus v6.5.9.3 (Hornbeck et al., 2015) and UniProt (Consortium, 2017). For each arginine of these proteins, we generated the 51-aa long sequence fragment with the central arginine. It is worth noting that if the central arginine is located at the N-terminus or C-terminus of the protein, the truncated sequence fragment will be padded with “_” to a length of 51 amino acid residues. The related sequence is defined as a positive sample if the central arginine is annotated as methylation. Otherwise, it is defined as a negative sample. We deduplicated the collected fragments. Accordingly, we collected 188,930 Arginine sites, including 9138 PRme sites and 179,792 non-PRme sites (Figure 1A). Because the number of PRme sites is only 5% of non-PRme sites, we randomly extracted 40,000 non-PRme sites as negative samples and considered the 9,138 PRme sites as positive and. We separated the dataset into a ten-fold cross-validation dataset (∼90%) and an independent test dataset (∼10%). The cross-validation dataset consisted of 8245 positive samples and 35,972 negative samples, and the independent test dataset included 893 positive samples and 4028 negative samples.

### 2.2 Feature encoding schemes

To create a methylated arginine predictor with high performance, we employ 19 feature encoding schemes, introduced below.

In the one-Hot encoding scheme (Wang et al., 2017), each amino acid is defined as a 20n length vector. Since only one of the 20 bits is 1, it uniquely represents the twenty amino acids. The rest feature encoding approaches (Chen et al., 2018b) include Dipeptide deviation from the expected mean (DDE), dipeptide composition (DPC), Enhanced Amino Acid Composition (EAAC), Composition of k-spaced Amino Acid Pairs (CKSAAP), Distribution (CTDD); Enhanced GAAC (EGAAC), Transition (CTDT), Composition of k-Spaced Amino Acid Group Pairs (CKSAAGP), Conjoint Triad (CTriad), k-Spaced Conjoint Triad (KSCTriad), binary encoding (BINA), grouped tripeptide composition (GTPC), BLOSUM62, Composition (CTDC), grouped dipeptide composition (GDPC), Z-Scale (ZSCALE), amino acid composition (AAC) and Grouped Amino Acid Composition (GAAC).

### 2.3 Model construction

We constructed machine-learning models using seven algorithms such as Decision Tree Classifier (Strobl et al., 2009), Gaussian NB (Huang and Hsu, 2002), k-nearest neighbours (Gil-Pita and Yao, 2008), Bagging Classifier (Dong et al., 2006), Random Forest (Pang et al., 2006) (Pang et al., 2006), Logistic Regression (Sperandei, 2014), and SVC (Cai et al., 2003). Their default parameters were used for development, using the corresponding packages in the sklearn of python3 (Supplementary Table S1). We also used Convolutional Neural Network (CNN) algorithm to build deep-learning models (parameters listed in Supplementary Table S2). Each algorithm is described briefly below.

#### 2.3.1 Random forest

The Random Forest classifier is an ensemble of multiple decision tree classifiers, each of which is trained from a different training set and features (Pang et al., 2006).

#### 2.3.2 Support vector classifier (SVC)

SVM is one of the most robust prediction methods based on statistical learning frameworks or VC theory (Cai et al., 2003). Given a set of training examples, each marked as belonging to one of two categories, and an SVM training algorithm builds a model that assigns new examples to one category or the other, making it a non-probabilistic binary linear classifier (although methods such as Platt scaling exist to use SVM in a probabilistic classification setting). An SVM maps training examples to points in space to maximize the width of the gap between the two categories. New examples are then mapped into the same space and predicted to belong to a category based on which side of the gap they fall.

#### 2.3.3 K-Nearest Neighbours algorithm

K-Nearest Neighbours algorithm is a statistical classifier that calculates the distance between the data features to be classified and the training data features and sorts them, takes out the K training data features with the closest distance; then determines the new sample category according to the category of the K similar training data features: if they all belong to the same category, then the new sample also belongs to this category; otherwise, each candidate category is scored, and the category of the new sample is determined according to a specific rule (Gil-Pita and Yao, 2008).

#### 2.3.4 Gaussian NB

Bayes Theorem describes the probability of an event based on prior knowledge of conditions related to the event. Gaussian Naive Bayes is one classifier model that assumes that the prior probability of a feature is usually distributed (Huang and Hsu, 2002).

#### 2.3.5 Decision tree classifier

The decision tree model is a tree structure; each internal node represents a test on an attribute, each branch represents a test output, and each leaf node represents a category. When running, using training data to establish a decision tree model based on the principle of minimizing the loss function; and when predicting, using the decision tree model to classify new data. It includes three steps: feature selection, decision tree generation, and decision tree pruning (Strobl et al., 2009).

#### 2.3.6 Bagging classifier

The bagging algorithm is representative of parallel integrated learning, which is mainly divided into four steps 1) cleaning the data according to the actual situation; 2) random sampling: repeat T times and randomly select T sub-samples from the sample each time; 3) individual training: Put each sub-sample into individual learner training; 4) classification decision: Use voting method integration to make the classification decision (Dong et al., 2006).

#### 2.3.7 Logistic regression

It is the preferred method for binary classification tasks (Sperandei, 2014). It outputs a discrete binary result between 0 and 1. Moreover, logistic regression measures the relationship between the dependent variable (the label we want to predict) and one or more independent variables (features) by using its inherent logistic function to estimate probability. These probabilities need to be binarized. The task of the logistic function is also known as the sigmoid function, which is an S-shaped curve. It can map any real value to a value between 0 and 1, but it cannot be 0 or 1. Then use a threshold classifier to convert values between 0 and 1 to 0 or 1. Maximum likelihood estimation is a general method for estimating parameters in statistical models.

#### 2.3.8 The deep-learning CNN algorithm

**FIGURE 2:**
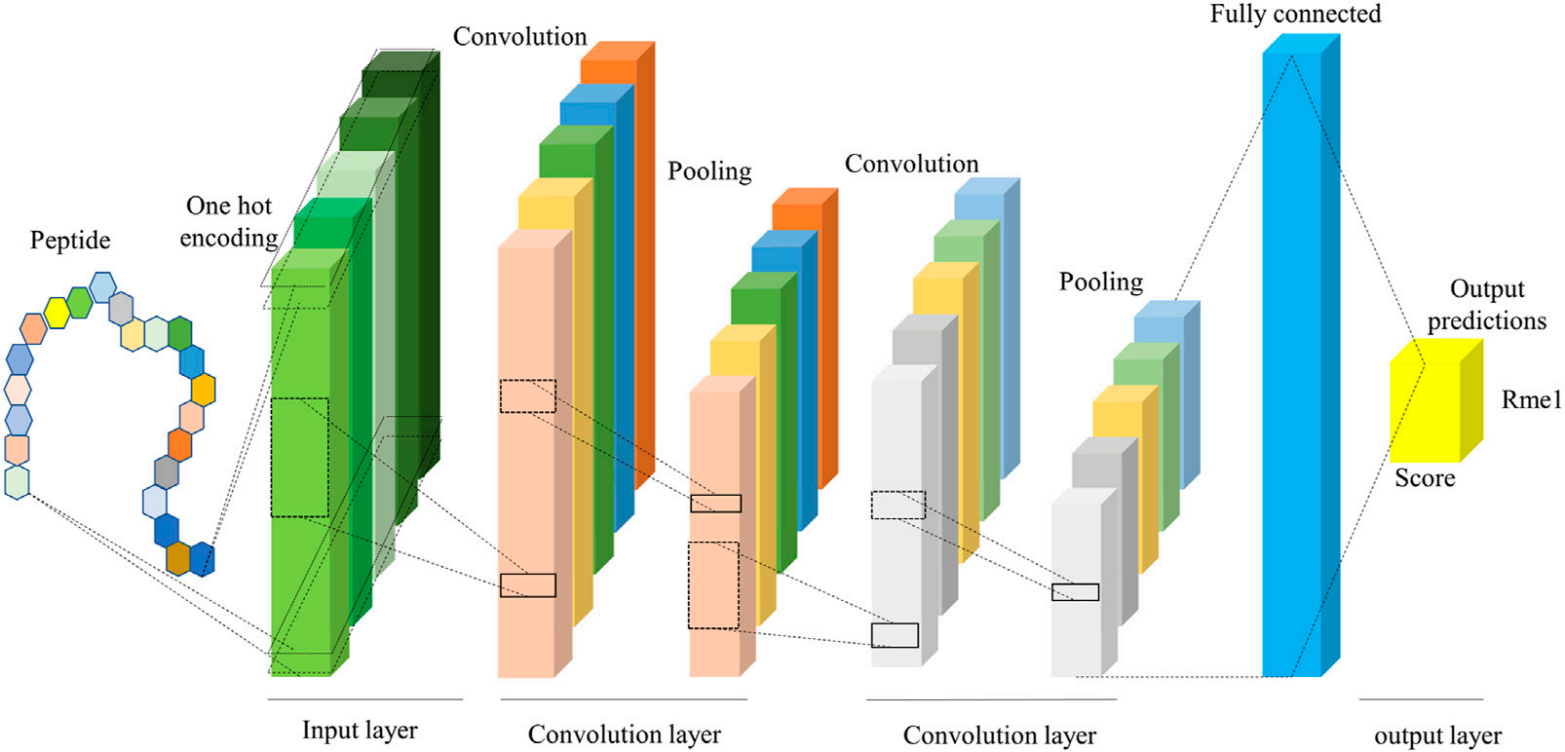
The framework of the CNN model. It includes input, convolution, and output layers, where the convolution layer extracts the features from the peptide sequence.

Deep learning is a sub-discipline of machine learning. Deep learning is based on artificial neural networks with representation learning that aim to mimic the human brain. The key difference between deep learning and traditional machine learning algorithms such as support vector machine (SVM) and random forests (RF) is that deep learning can automatically learn features and patterns from data without handcrafted feature engineering (Wen et al., 2020). We took the 1D-CNN Model with One-Hot encoding (CNN_OH_) as an example to illustrate the deep-learning network framework. This model contains four layers, listed below (Figure 2).

1. Input layer. The One-Hot encoding encodes each input sequence of 51 amino acids to a 51 × 21 binary matrix.
2. Convolution layer. It consisted of two convolution sublayers, each followed by a max-pooling sublayer. The first convolution sublayer includes 256 different convolution kernels with a size of 9 × 21. Each kernel is applied to the 51 × 21 matrix and results in a feature vector with the size of 43 (= 51–9+1). Thus, the 256 kernels output a 43 × 256 matrix. Next, a pooling kernel with the size of 2 is applied to the feature matrix and produces a 21 × 256 matrix. In the second convolution sublayer, 32 different convolution kernels with the size 7 × 256 are applied to generate a 15 × 32 matrix, followed by a pooling kernel with size two that produces a 7 × 32 data matrix.
3. Fully connected layer. The 7 × 32 data matrix generated from the convolution layer is nonlinearly transformed to 128 representative features.
4. Output layer. The modification score is calculated based on the 128 features using the “Sigmoid” function.

### 2.4 Model training

To avoid overfitting, we use early stopping, a widely used method to screen the better models, and use the cross-validation method to get the best prediction model by integrating all the better models.

### 2.5 Performance evaluation

To evaluate the performance of models, we used Sensitivity (Sn), Specificity (Sp), and the area under the Receiver Operating Characteristic (AUC) as the performance metrics. Sn defines the model’s ability to identify positive residues from actual positive residues; the Sp measures the model’s ability to identify the negative samples from the actual negative samples; AUC measures the comprehensive performance of the model.

### 2.6 Statistical methods

The paired student’s t-test was used to test the significant difference between the mean values of the two paired populations. The threshold is set to 0.05.

### 2.7 GO and KEGG analysis

Gene Ontology (GO) analysis for enriched “biological process” terms and enriched genes in KEGG pathways were performed using R (v4.0.4), including clusterProfiler, topGO, org. Hs.eg.db, AnnotationDbi, stats4, BiocGenerics, Iranges, and enrichplot packages.

## 3 Results

### 3.1 The CNN-based model performed better than traditional machine-learning-based models

**FIGURE 3:**
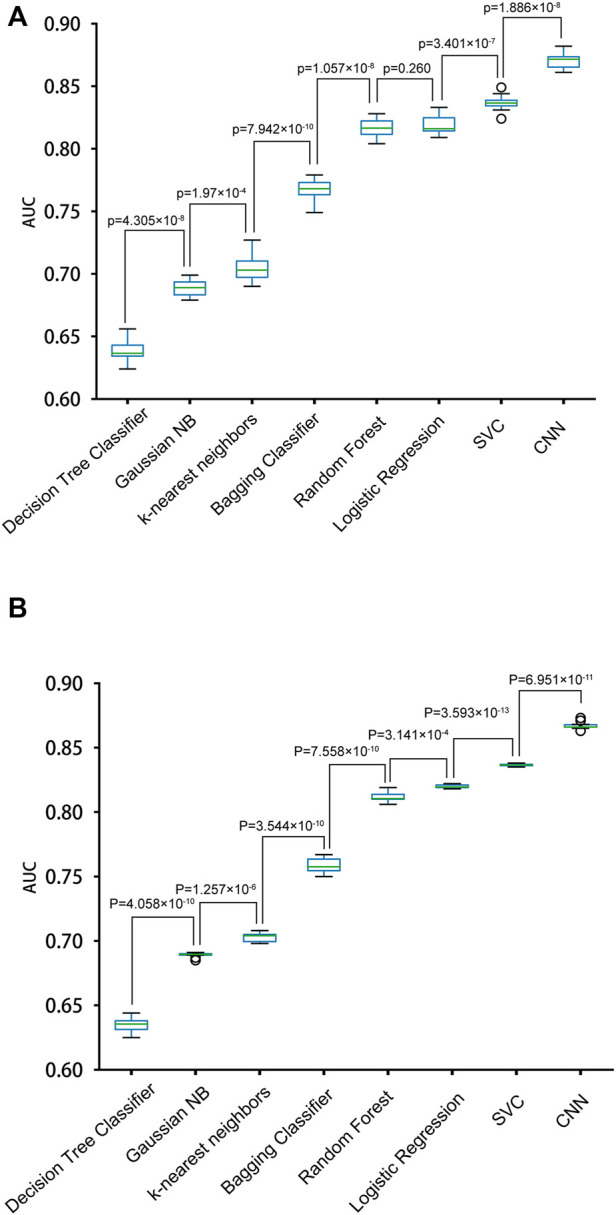
Performances of different models with the One-Hot feature for predicting PRme sites. **(A)** The AUC values of different models in ten-fold cross-validation. **(B)** The AUC values of different models in the independent test.

We constructed eight prediction models by integrating seven machine-learning algorithms and the CNN algorithm with the simple One-Hot encoding approach and compared their performances. The seven machine-learning algorithms included Random Forest, SVC, K-nearest Neighbors Classifier, Gaussian NB, Decision Tree Classifier, Bagging Classifier, and Logistic Regression. The result metrics (AUC, Sn (Sp = 0.9), Sn (Sp = 0.95), Sn (Sp = 0.99)) were used for evaluation in the ten-fold cross-validation and the independent test (Table 1 and Figure 3). The average AUC value and the Sn values of CNN_OH_ model were the largest among the eight models. Therefore, CNN_OH_ is the best model and has excellent predictive ability. Additionally, among the machine-learning models, the average AUC values of SVC, Logistic Regression and Random Forest algorithms were 0.8367, 0.8189, and 0.8167, respectively, which were the three best machine-learning models.

**TABLE 1:** Prediction Performances of different models integrating the One-Hot encoding approach.

Performances in ten-fold cross-validation				
	AUC	Sn (Sp = 0.9)	Sn (Sp = 0.95)	Sn (Sp = 0.99)
Gaussian NB	0.6884	0.4579	0	0
Decision Tree Classifier	0.6383	0.4143	0	0
K-nearest Neighbors	0.7047	0.3149	0.3149	0.1061
Bagging Classifier	0.7678	0.5049	0.3845	0.1554
Random Forest Classifier	0.8167	0.5538	0.4234	0.2144
Logistic Regression	0.8189	0.5332	0.3925	0.1706
SVC	0.8367	0.5812	0.4355	0.2085
CNN	0.8708	0.6642	0.5174	0.2231
Performances in the independent test				
	AUC	Sn (Sp = 0.9)	Sn (Sp = 0.95)	Sn (Sp = 0.99)
Gaussian NB	0.6891	0.4528	0	0
Decision Tree Classifier	0.6347	0.409	0	0
K-nearest Neighbors	0.7029	0.314	0.314	0.1136
Bagging Classifier	0.7583	0.4943	0.3567	0.1477
Random Forest Classifier	0.8121	0.5345	0.4131	0.2165
Logistic Regression	0.8199	0.5255	0.3757	0.1409
SVC	0.8365	0.5756	0.4206	0.1929
CNN	0.8671	0.6538	0.5025	0.1902

### 3.2 Performance comparison of models with different encoding approaches

**FIGURE 4:**
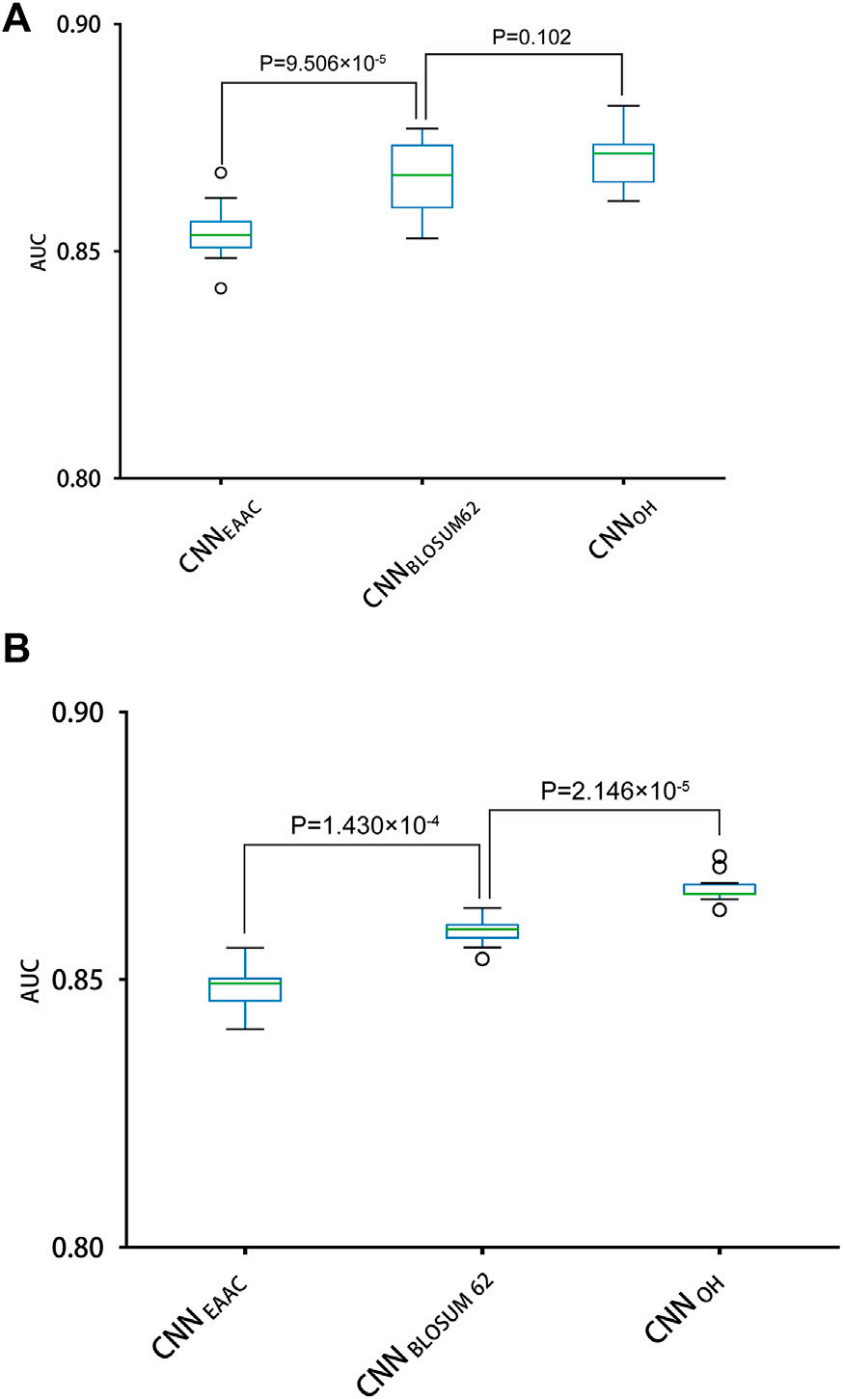
Performances of the CNN models with different features for predicting PRme sites. **(A)** The AUC values of different models using ten-fold cross-validation. **(B)** The AUC values of different models in the independent test.

With the One-Hot encoding approach, we found that the CNN algorithm and three machine-learning algorithms (i.e., SVC, Logistic Regression and Random Forest) had the highest performances compared to others. To evaluate the effect of encoding approaches on the prediction performance, we collected 17 other encoding approaches and compared them with the One-Hot approach, integrated with the three best machine-learning algorithms (see methods for details). Table 2 summarizes the AUC values of these models in terms of ten-fold cross-validation. It can be seen that the machine-learning models with three encoding approaches (i.e., BLOSUM62, One-Hot and EAAC) achieved the largest AUC values. Accordingly, we constructed CNN models using the three encoding approaches. Figure 4 shows that the average AUC value of CNN_OH_ is statistically larger than those of CNN_EAAC_ and CNN_BLOSUM62_ in the independent test, although CNN_OH_ and CNN_BLOSUM62_ had similar AUC values in ten-fold cross-validation. Based on these observations, we chose CNN_OH_ as the predictor of arginine methylation and named it CNNArginineMe.

**TABLE 2:** Prediction performances of the models integrating different algorithms and various feature encoding approaches in ten-fold cross-validation.

	Random forest	SVC	Logistic regression	CNN
GAAC	0.606	0.609	0.566	
GDPC	0.71	0.658	0.635	
GTPC	0.736	0.667	0.693	
CTDD	0.718	0.693	0.692	
CKSAAGP	0.737	0.676	0.699	
CTDT	0.734	0.709	0.696	
KSCTriad	0.74	0.699	0.726	
CTriad	0.745	0.699	0.726	
EGAAC	0.736	0.713	0.731	
CTDC	0.756	0.72	0.704	
AAC	0.775	0.725	0.701	
ZSCALE	0.805	0.738	0.772	
DPC	0.804	0.759	0.798	
DDE	0.801	0.766	0.798	
CKSAAP	0.8	0.78	0.801	
BLOSUM62	0.807	0.821	0.834	0.848
One-Hot	0.813	0.839	0.822	0.871
EAAC	0.82	0.819	0.841	0.859

### 3.3 Comparison of CNN_OH_ with reported predictors

**FIGURE 5:**
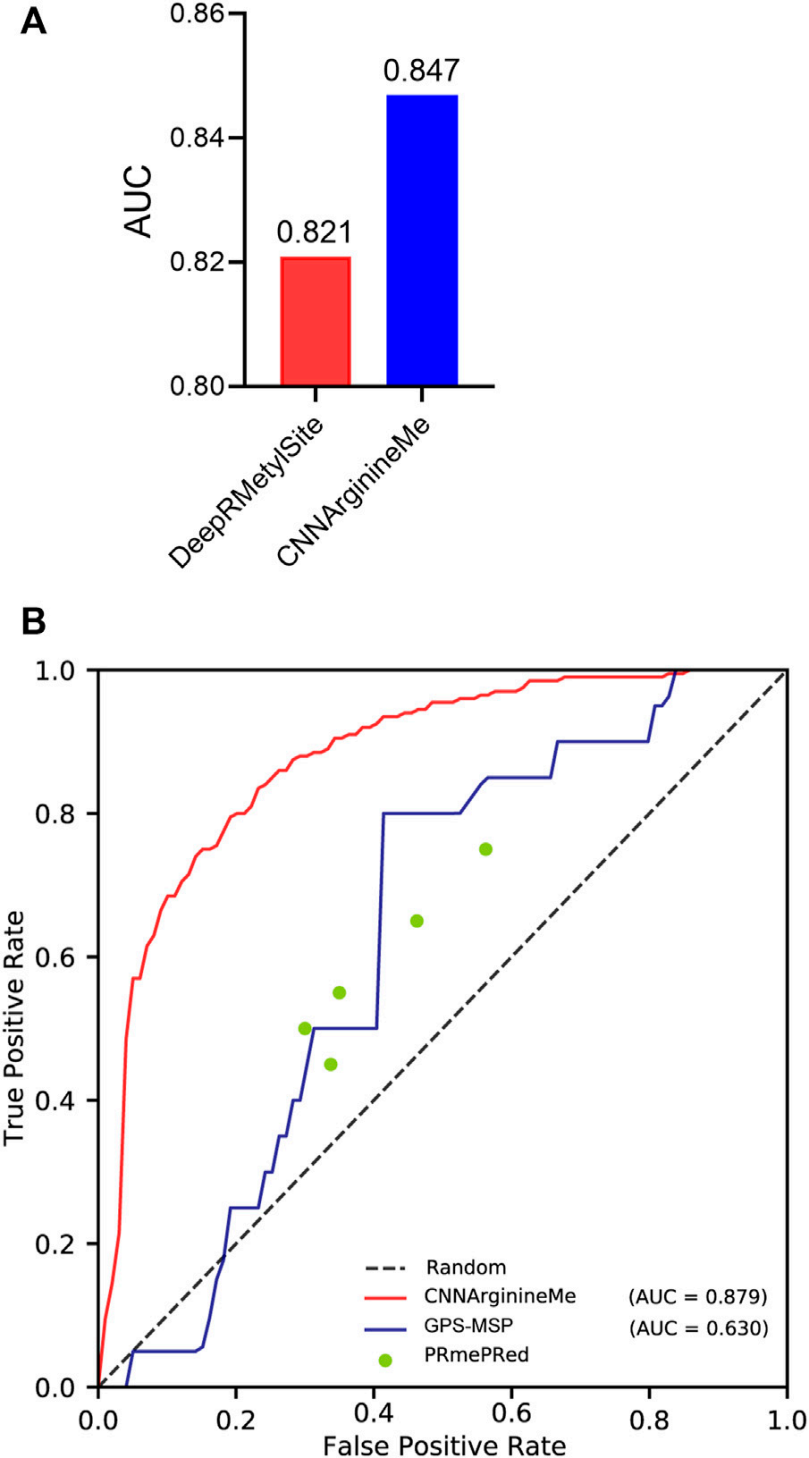
The comparison of CNNArginineMe and reported classifiers. **(A)** AUC values of reconstructed CNNArginineMe and DeepRMethylSite. **(B)** The comparison between CNNArginineMe and two reported online prediction models, i.e., GPS-MSP and PRmePRed.

To examine the predictive quality of the proposed CNNArginineMe, we compare it with reported PRme site predictors. DeepRMethylSite is the latest deep-learning predictor with the best performance compared to other reported ones (Chaudhari et al., 2020). To fairly compare CNNArginineMe and DeepRMethylSite, we used the dataset to construct DeepRMethylSite to rebuild CNNArginineMe and employed its independent test set for evaluation. Figure 5A shows that the AUC value of CNNArginineMe is 0.847, which is higher than that (0.821) of DeepRMethylSite. Furthermore, we selected two more reported predictors developed recently that provide available online prediction websites for comparison, i.e. PRmePRed (Kumar et al., 2017) and GPS-MSP (Deng et al., 2017). Due to the upload limit of the online websites, we randomly 100 sequences from our independent dataset, where the proportion of positive samples was the same as that in the independent test set. We used these 100 sequences to benchmark the three classifiers. Figure 5B shows that CNNArginineMe had the best performance among these models. Therefore, CNNArginineMe has outstanding performance for predicting PRme sites.

### 3.4 Prediction and functional analysis of arginine methylated proteome

**FIGURE 6:**
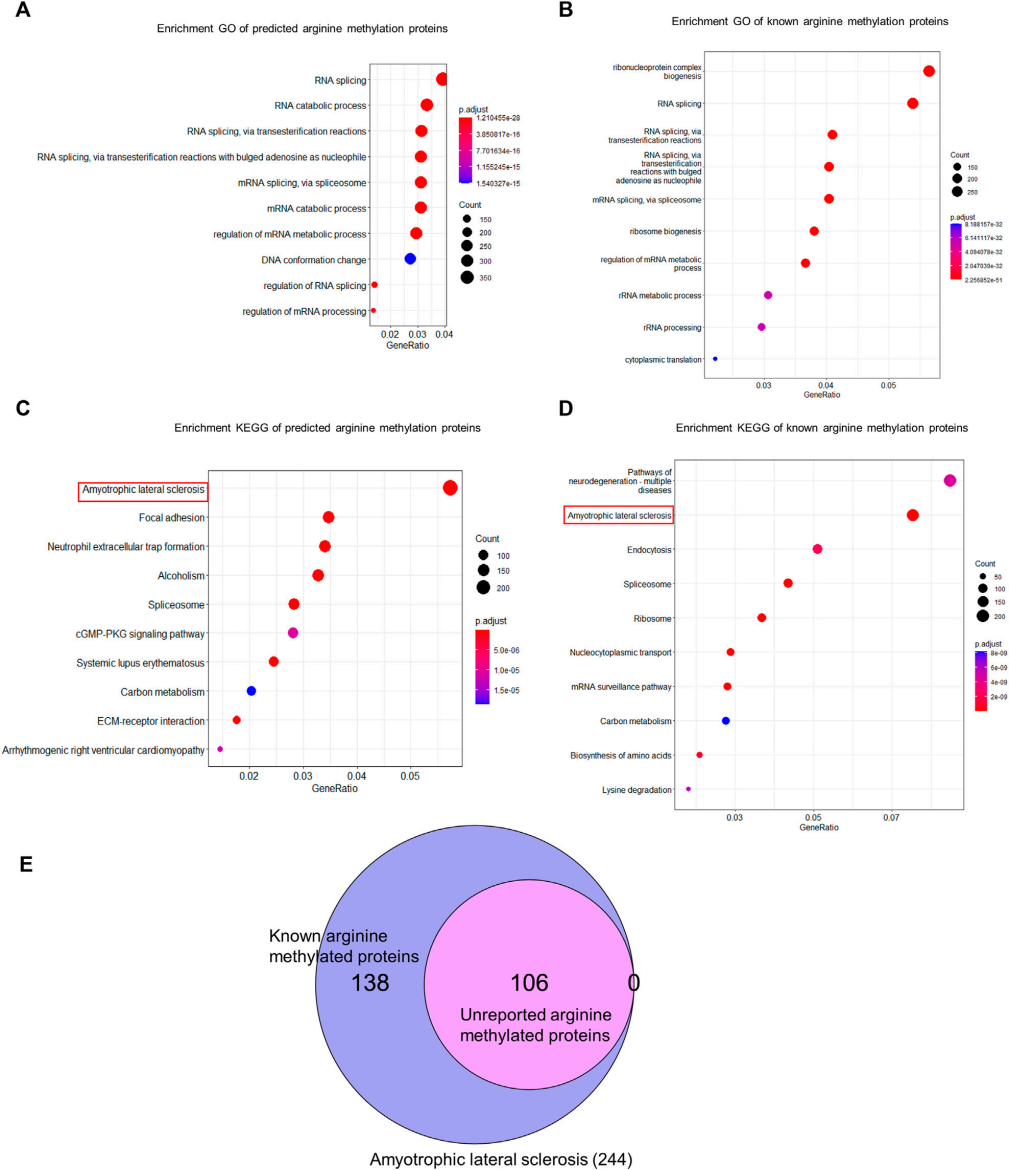
GO and KEGG analysis of human arginine methylated proteome. **(A)** GO enrichment analysis of arginine methylated proteome predicted by CNNArginineMe. **(B)** GO Enrichment analysis of known arginine methylated proteins. **(C)** KEGG enrichment analysis of arginine methylated proteome predicted by CNNArginineMe. **(D)** KEGG enrichment analysis of known arginine methylated proteins. **(E)** Venn Diagram of reported and predicted arginine methylated proteins involved in the ALS.

We used CNNArginineMe to predict PRme sites from human proteome with the threshold value corresponding to the specificity of 0.95. We predicted 47888 PRme sites from 19023 proteins, most of which have not been reported. We performed functional analysis of the predicted arginine methylated proteome using Gene Ontology and the KEGG pathway. The GO enrichment analysis showed that arginine methylated proteome is enriched in RNA splicing, RNA catabolic process, mRNA splicing, mRNA catabolic process, and regulation of mRNA metabolic process (Figure 6A). It is similar to the enrichment of known PRme-containing proteins (Figure 6B). Moreover, based on the KEGG pathway, the predicted arginine methylated proteome is significantly enriched in amyotrophic lateral sclerosis (ALS) (Figure 6C). This same observation could be made for reported PRme-containing proteins (Figure 6D). The ALS pathway contains 244 proteins, of which 106 without methylation annotation were predicted by CNNArginineMe (Figure 6E). According to the Amyotrophic Lateral Sclerosis Online Database, 154 proteins are linked to ALS, and 16 of them are predicted to be arginine methylated (Table 3) (Abel et al., 2012; Yun and Ha, 2020). Out of the 16 proteins, four (i.e., ATX2, FUS, ROA1, and TADBP) contain known PRme sites (Rappsilber et al., 2003; Ong et al., 2004; Guo et al., 2014). Moreover, six of the 16 proteins (i.e., ANXA11, FUS, HNRNPA2B1, HNRNPA1, TARDBP, and VAPB) have common genetic mutations in ALS, suggesting that these mutations may affect arginine methylation (Kabashi et al., 2008; Kim et al., 2013; Picchiarelli et al., 2019; Cadoni et al., 2020; Nahm et al., 2020). In summary, the arginine methylated proteome predicted by CNNArginineMe has similar enrichment features to the known arginine methylated proteins, which may assist the understanding of the functions of arginine methylation.

**TABLE 3:** Number of PRme sites in the ALS-related proteins.

Uniprot ID	Protein name	Number of predicted PRme sites	Number of known PRme sites
Q9Z269	VAPB	2	0
P09651	ROA1	11	8
P12036	NFH	5	0
P22626	ROA2	12	0
P35637	FUS	29	22
P41219	PERI	10	0
P50995	ANX11	3	0
P68366	TBA4A	2	0
Q13148	TADBP	2	1
Q14203	DCTN1	17	0
Q15303	ERBB4	3	0
Q53GS7	GLE1	1	0
Q96CV9	OPTN	3	0
Q96JI7	SPTCS	1	0
Q99700	ATX2	26	1
Q9UMX0	UBQL1	9	0

## 4 Discussion and conclusion

Many classifiers for predicting various types of PTM sites have been developed by integrating machine-learning or deep-learning algorithms with different encoding features (Chen et al., 2018a; Huang et al., 2018; Chen et al., 2019; Lyu et al., 2020; Zhang et al., 2020; Zhao et al., 2020; Sha et al., 2021; Wei et al., 2021; Zhu et al., 2022). It has been found that the models based on deep-learning algorithms have better prediction performances than those based on traditional machine-learning algorithms. The same observation is also made in this study (Table 2). The CNNArginineMe model integrating the CNN algorithm and the One-Hot encoding approach compares favourably to the machine-learning models integrating distinct algorithms and various encoding features (Table 2). These observations indicate that deep-learning algorithms must be prioritized during model construction to predict PTM sites. In this study, we compared CNNArginineMe with three reported classifiers for predicting PRme sites, i.e., DeepRMethylSite, GPS-MSP and PRmePRed. CNNArginineMe shows superior performance. It may be due to several reasons. Firstly, our dataset for model construction is relatively large, and a deep-learning model with excellent performance requires big data. Secondly, the early stop strategy is used for model construction to avoid overfitting. Nevertheless, CNNArginineMe fails to distinguish between different PRme types. Shortly, we will develop new models for predicting PTM sites with different PRme types.

We used CNNArginineMe to predict arginine methylated proteome and performed GO and KEGG analyses to understand the role of arginine methylation. Our results show that critical proteins of ALS are highly arginine methylated, implying that ALS is related to arginine methylation. Besides, arginine methylation is related to RNA splicing. This observation is consistent with the reports that gene expression is activated or repressed by arginine methylation (Fulton et al., 2019), and splicing fidelity is reduced by inhibiting symmetric or asymmetric demethylation of arginine, mediated by PRMT5 or type I PRMTs (Fong et al., 2019).

In summary, accurate identification of PRme sites could be effective in deciphering the functional and structural characteristics of protein methylation that plays an essential role in cell biology and disease mechanisms, and it will help understand transcriptional regulation, RNA splicing, DNA damage repair, cell differentiation, and apoptosis (Al-Hamashi et al., 2020).

## Data Availability

Publicly available datasets were analyzed in this study. This data can be found here: https://github.com/guoyangzou/CNNArginineMe.
