# Supplementary material for: CNNArginineMe: A CNN structure for training models for predicting arginine methylation sites based on the One-Hot encoding of peptide sequence
Source: Front Genet. 2022 Oct 17;13:1036862. doi: 10.3389/fgene.2022.1036862 (PMC9618650; doi:10.3389/fgene.2022.1036862)
Supplement: Supplementary file 1 [file DataSheet1.docx]

CNNArginineMe: a CNN structure for training models for predicting arginine methylation sites based on the One-Hot encoding of peptide sequence

Jiaojiao Zhao^1,2#^, Haoqiang Jiang^2#^, Guoyang Zou^2^, Qian Lin^1^, Qiang Wang^3^, Jia Liu^4^, Leina Ma^1*^

**Supplementary Tables**

**Table S1. The methods and default parameters used for developing machine- learning models in Sklearn library.**

| Name | importing method | creating method |
| --- | --- | --- |
| Random Forest Classifier | From sklearn.ensemble import Random Forest Classifier | model = Random Forest Classifier () |
| SVC | from sklearn.svm import SVC | model = SVC (probability=True) |
| K-nearest Neighbors Classifier | from sklearn.neighbors import K-Neighbors Classifier | model = K-Neighbors Classifier () |
| Gaussian NB | from sklearn.naive_bayes import Gaussian NB | model = Gaussian NB () |
| Decision Tree Classifier | from sklearn.tree import Decision Tree Classifier | model = Decision Tree Classifier () |
| Bagging Classifier | from sklearn.ensemble import Bagging Classifier | Model = Bagging Classifier () |
| Logistic Regression | from sklearn.linear_model import Logistic Regression | model = Logistic Regression (max_iter=1000) |

**Table S2. Parameters of the single-task CNN model.**

| layer | option | value |
| --- | --- | --- |
| Cov 1D | filter number | 256 |
|  | kernel size | 9 |
|  | stride | 1 |
|  | padding | valid |
|  | batchnormalization | yes |
|  | activation | relu |
|  | maxpool | 2 |
|  | dropout | 0.7 |
| Cov 1D | filter number | 32 |
|  | kernel size | 7 |
|  | stride | 1 |
|  | padding | valid |
|  | batchnormalization | yes |
|  | activation | relu |
|  | maxpool | 2 |
|  | dropout | 0.5 |
| Dense | size | 128 |
|  | activation | relu |
| Dense (output) | size | 1 |
|  | activation | sigmoid |
